# Researching and practicing ARTIVISM through field-crossing: an innovative method for collaborative research

**DOI:** 10.12688/openreseurope.13634.1

**Published:** 2021-09-13

**Authors:** Monika Salzbrunn

**Affiliations:** 1 FTSR-ISSR-Anthropole 5067, University of Lausanne, Lausanne, Vaud, 1015, Switzerland

**Keywords:** Field-crossing, collaborative research, qualitative methods, ethnography, ethics, reflexivity, anthropology, sociology

## Abstract

While collaborative research has mainly focused on the relationship between researchers and research partners in the field, several recent works have contributed to reflecting on the relationships between researchers of various backgrounds and roles, and the challenges and benefits of teamwork. While conducting the ERC-funded ARTIVISM project, I have continued to rethink the ways research can be conducted collectively, especially when starting from a multi-sensory approach and by applying apprenticeship and audio-visual techniques. I developed the method of field-crossing, which allows the researcher to regularly contrast perspectives and perceptions, and which helps researchers regain emotional and intellectual independence after an intensive, year-long period of fieldwork as and among artivists. The article shows how field-crossing allows its practitioners to reflect in an innovative way on their positioning in a field, open new perspectives, integrate surprises and disruptive and unexpected developments, and cope with inner and collective conflicts. Finally, collective feedback sessions about text and image publications (comics, films) with the artivists are not only part of our ethical approach but also serve as elicitation sessions which reflect the complexity of field relations and individual and collective perspectives among and between field-crossers and artivists.

## Introduction

Since its beginnings, anthropological research has navigated the space between the discovery of new fields and subjects, and the constant follow-up research of familiar territories throughout a researcher’s career. How can we retain our capacity to view new developments in a seemingly familiar field? How can we nourish our capacity to let ourselves be surprised by unexpected, seemingly incomprehensible events? How can we deal individually and collectively with disruptive events that occur within planned events? The event approach ( Salzbrunn, 2021), applied to the research of artivism, is a constructive answer to groupism ( Brubaker, 2004), but requires the highest adaptability of researchers to the unexpected. How can we deal with biased perceptions of the field, its actors, and ourselves? The need to remain curious and open is not only a challenge in long-term studies abroad, but also in new fields or innovative subjects explored next door. Hirschauer & Amann (1997) have suggested several techniques for achieving the alienation of our own culture, that is for fostering a reflective, fresh perspective on seemingly familiar environments. Working in an environment which is encompassed by the researcher’s indigenous knowledge requires a very high degree of reflexivity during the mental and emotional distancing process. Below, I will develop more in detail the benefits of field-crossing in this process.

In the present article, I will outline the method of field-crossing as a new form of collaborative research which I created and practiced over the last ten years in teaching and research. A field-crosser unfamiliar with the area and/or the subject joins the main researcher in her/his field and allows the latter to benefit from her/his questions, surprises, and misunderstandings, in order to open new perspectives and to improve reflexivity when leaving the field after an immersive process. I began employing this method in Japan and France with my colleague professor Yasumasa Sekine and am currently testing and developing the approach with the help of my ERC ARTIVISM team (senior researcher Raphaela von Weichs, PhD students Federica Moretti and Sara Wiederkehr) in Cameroon, Italy, France and California. The current project focuses on “Art and activism. Creativity and Performance as Subversive Forms of Political Expression in Super-Diverse Cities” and seeks to “explore new artistic forms of political expression under difficult, precarious and/or oppressive conditions”…“Going beyond former research in urban and migration studies, and beyond the anthropology of art, ARTIVISM focuses on a broad range of artistic tools, styles and means of expression, namely festive events and parades, cartoons and comics and street art” ( Salzbrunn, 2015:1). Before elaborating on field-crossing in general and in the context of ARTIVISM in particular, I will give an overview of collaborative methods in anthropology and related current debates.

### What do teamwork and collaborative research mean in anthropology and qualitative sociology?

Bibliographical research on collaboration in social sciences in English, French and German shows that collaboration mostly concerns the ways researchers and the researched – considered as active co-constructors of the field – work together (see e.g. Pardee *et al.,* 2018). A recent issue of *SociologieS*, the journal of the International Association of French-speaking Sociologists (AISLF), is dedicated to ethical issues in collaborative research. Larouche *et al.* (2020) have edited several articles dealing with the ambiguous ethical assumption that a well-prepared and reflexive collaboration between researchers and research subjects can be democratic, fair, and equal. However, this commonly assumed epistemological point of departure leads in certain cases to control or even censorship of research outcomes, which cannot be published in case the final results do not correspond to the self-perception of the research participants, as Jean-Michel Chaumont shows in his contribution on mistrust in collaborative research (2020). The question of how to balance the right of research participants to express their opinion, up to the point of vetoing research results, with the need to preserve and protect the independence of research has been discussed by many academic institutions and researchers (see Lavanchy and others in the Swiss Society for Ethnology 2011 and the related journal Tsantsa 2018). Furthermore, the construction of ethnographic knowledge has considerably evolved, as George E. Marcus notes:

The results of ethnographic research today are less clear, certainly more specific, and indeed more ethnographic in quality. This means that ethnographic knowledge creates itself in parallel with and relation to similar functions in the very communities which it makes its subjects. This leads to the more urgent need for modalities of collaboration as method, already mentioned, but also to mediation and intervention as being the primary form and function of the knowledge that ethnography produces ( Marcus, 2010: 275).

Hence, the relationship between researchers and participants is part of an ongoing dynamic within the field and beyond, namely during the processes of restitution, publication, and dissemination, which remain understudied. A current and well-thought-out example of collaborative research that integrates these methodological and ethical reflections is the Franco-German Migreval program with its CollabMigr project ( Évaluation biographique des politiques par les migrants en Europe), led by professors Catherine Delcroix and Ursula Apitzsch ( Apitzsch & Delcroix, (dir.)), in which recorded life-stories are shared and interpreted collectively, and their interpretation restituted to the interviewees. This project integrates a reflection on the nature of collaboration between researchers and interviewees as well as among researchers. A large number of the researchers who collaborate on the interpretations of the interviews during CollabMigr have not been part of the same team or project, so that the contextualization is provided by the main researcher. I will now further examine the question of collaboration amongst researchers as a long-term process within a team.

In their work on “Cultures in the making”, Chistina Siry, Caroline Ali-Khan, and Mark Zuss (2011) aim to set up experimental, lived and qualitative relations within "emergent research communities":

We believe that the relations between researchers themselves, the supposedly "horizontal" level of traditional fieldwork, must be brought to constant reflection and negotiation if it is to adhere to truly participatory principles of collaboration. If researchers who work together do not commit to rigorous reflection, and to revisiting this commitment to rigor, (individually as well as collectively) then we contend that they (we) run the risk of slipping into unequal and perhaps inequitable power relationships. We have noticed how the everyday practices of "getting things done" in research can push against the notion of equitable collaboration. In addition, as personality dispositions steer us, and friendships compel us, the nature of any collaboration becomes increasingly complex. ( Siry *et al.*, 2011:2)

Being aware of the unavoidable aspect of power relations among researchers, between the subjects in the field, and within the merged situational community of artivists which emerges during fieldwork, I argue that constant reflexivity is the best way to remain conscious of these dynamics. In order to cultivate this state of mind individually and collectively, from the conception of the project, during fieldwork and analysis, through restitution, publication, and dissemination, I have developed the method of field-crossing. During field-crossing, this process of reflection is undertaken and conducted in a relational way with other researchers as part of the whole process. Besides the important ethical dispositions cited above, field-crossing also helps to gain an unconventional access to the field – to better see, perceive, and feel what is going on and, intermittently, to find one’s way out of a multi-sensory immersion, and back again, in order to gain feedback from the researched about the research output. As I will show later, the discussions between the main researcher and the field-crosser also contribute a great deal to (re)gaining distance and adopting a critical view on the past and ongoing dynamics within the field – especially in an emotionally charged environment. Wider political events, any personal conflicts which may arise, emergent friendships or mutual engagement for a cause can all affect the participants. It is therefore important to reflect together on the impact emotions have had on each researcher’s relation to the field and her findings. Taking into consideration how fieldwork affects each individual via field-crossing is part of the affective turn in social sciences (after a long period influenced by positivism and scientism) and contributes to the further development of multi-sensory anthropology.

### Getting started: access to the field

Peter Rieker, Giovanna Hartmann Schaelli, & Silke Jakob have recently (2020) demonstrated the complexity of access to the field, which has to be constantly negotiated and which evolves over time. Positioning within the field is related to the perception of the team members’ multiple aspects of belonging (gender, age, family members, way of life, origin, language abilities, political attitude/opinion, attitude towards religion /practice etc.) and the hierarchy within the research team. Because, for example, PhD students gain different access to the field as professors or senior researchers, contrasting both field journals, impressions and feelings, considerably enriches the findings.

Furthermore, a distinction can be made between researchers who are already part of a group or milieu and decide to carry out research on it, and those who need to enter a field as researchers ( Becker, 1963; Touraine, 1965; Wacquant, 2000) and/or actively engaged members/apprentices. Those who are familiar with a milieu and are active group members need to reflect intensively on the ways they shift roles, mentally and physically, publicly and to a certain extent privately. The others need to carefully prepare the way they enter a new field ( Ballon *et al.,* 2019; Rieker *et al.,* 2020), coping with fears ( Devereux, 1980), uncomfortable situations ( Caratini & Godelier, 2012; Williams, 2012), unexpected evolutions, but also enthusiasm and excitement which characterise the opening of a new field.

Ethical considerations are obviously important for each case: Is undercover research necessary to gain access to a specific field (such as the police, see Palidda, 2015, or other delicate milieus and situations, see Lavanchy, 2012 and Avanza, 2008), and/or can undercover practice be justified for a researcher who is known as a group member in a specific field, but not as a researcher who could use any information gathered during his or her leisure activities? Current debates on ethics include such important questions ( Lavanchy, 2012; Iphofen & Tolich, 2018), which we will expand upon elsewhere ( Salzbrunn & von Weichs, in preparation). Field-crossing offers a solution for coping with the difficulties of entering, being in and leaving a field, thanks to reflexive collaborations among researchers implicated on different levels. Gathering and discussing various points of view concerning the research conditions in an atmosphere of trust and sharing can bring to light difficulties that all too often remain taboo in ethnographic fieldwork, helping researchers gather advice and develop solutions.

In my teaching experiences as well as in my research settings, I have chosen various ways to set up teams for fieldwork: Two or more colleagues familiar with the field and two who are unfamiliar with the field or, in most cases, one researcher who is familiar at least with the cultural context of the field and one who discovers almost everything (language, cultural and economic context, milieu etc.) and/or who is perceived as being a newcomer when introduced by the main researcher. The latter has turned out to be extremely productive for both sides in various previous research settings: During my research on Senegalese political and religious networks ( Salzbrunn, 2004), I often went with Senegalese peer PhD students to the field in Paris, Dakar, or New York. They benefited from answers to questions I could dare to ask, because of my supposed unfamiliarity with the milieu. Although, as a famous French expression about research subjects has it, you do not need to be Caesar to understand Caesar, some interlocutors assume that non-Caesars have a total ignorance of the Caesar milieu, and are willing to give detailed answers to supposedly naïve questions which Caesars could not ask. Nevertheless, not every Caesar has a complete knowledge about Caesar and his milieu. Therefore, it can be an advantage to be a novice and to access a field without previous knowledge, moral judgements, or societal constraints. Furthermore, the details a non-Caesar notices of an environment (clothes, body-language etc.) can be interesting for the Caesar researcher because they might have been too accustomed to these elements to perceive or notice them. In other cases, namely in a collaborative situation that unites a senior and a junior researcher, the latter can learn from observing how the senior enters the field, how they become familiar with it, deal with loyalties, conflicts, emotions, ethical aspects etc. In return, the senior can give their recommendations directly to the junior colleague after sharing with them certain experiences. Obviously, the senior can also meet challenges in their main field and benefit from the junior’s advice thanks to the latter's distant perspective.

Field-crossing is also an interesting teaching method, especially in an environment like the city of Lausanne where 47% of the population has a foreign passport. The majority of my students have at least one foreign-born parent, so that field-crossing in various milieus can be easily implemented. This social fact provides tremendous possibilities for field-crossing in the field of migration and super-diversity, because I can encourage the students to build cross-cultural teams for their seminar work. The question of belonging as a “culture of place” ( hooks, 2009) is particularly important in this context. As access to nationality and land is particularly difficult in Switzerland, specifically young people imagine other local forms of belonging. In this context, researchers not only need to develop empathy, but also have to create and/or perform at least temporary expressions of commonality ( Salzbrunn & Sekine, 2011) or belonging in order to become accepted. The method of apprenticeship ( Lave, 2011) allows researchers to be accepted in a milieu, and to learn the research participants’ skills through a bodily experience. I will show later how I combine apprenticeship, a multi-sensory approach, and visual anthropology with field-crossing.

In my ERC ARTIVISM research setting, field-crossing has been a central method from the beginning. As I have written in the proposal ( Salzbrunn, 2015:7), referring to Pink (2009):

Sensory ethnography requires the researchers to perceive and reflect not only on the visual, but also the tactile, auditive, olfactory and other senses in different cultural contexts. The tools used in sensory, audio-visual and digital ethnography are pertinent to the investigation of the research object of ARTIVISM. Audio-visual fieldwork in this context is ambitious and central to a qualitative understanding of the multisensory and embodied experience of both the actors and the ethnographers.

During the ERC ARTIVISM project, we have accessed the field through various methods. Drawing, while focusing on multi-sensory perceptions, was a key element since it forced each researcher to take the time necessary to get familiar with the local environment ( Salzbrunn & von Weichs, 2020). Drawing, as opposed to shooting photos, allowed researchers to avoid being taken for tourists in certain places. It allows to access the field since it gives a role to the researcher during the first contact phase, before potentially becoming an apprentice. Drawings are also a rich source of interpretation and elicitation during the research process and during restitution. This technique also allows field-crossers to enter the field without necessarily understanding the language and/or the context of the field. Multi-sensory perceptions of an environment evolve over space and time and vary from one researcher to the other. Therefore, it is particularly instructive to confront different multi-sensory perceptions through field-crossing and feedback sessions.

### Field-crossing and apprenticeship in Japan and beyond

In the ERC ARTIVISM project, each researcher investigated her main research field and, temporarily, her colleagues’ fields. According to the logistic possibilities and the personal situation of each team member, a small number of long stays or a higher number of shorter stays have been conducted. Certain members could spend roughly 18 months in total engaged in active research, including a preparatory phase, a one-year intensive stay, and a follow-up and feedback period. Field-crossing was particularly crucial at the end of an intensive immersive year with apprenticeship as a research practice. Having become part of the field as an artivist in the chosen urban space, the researcher could temporarily distance herself from or subsequently exit the field situation thanks to the colleagues who joined her. These colleagues’ views on the field and its dynamics were crucial for understanding the main researcher’s implication in the field, the general dynamics, power relations, the impact of the researcher’s presence on the field, etc. Field-crossing helped maintain a balance between the long-debated risks of “nostrification” (following a Malinowski-style immersion) and “othering” ( Said, 1978), as Breidenstein, Hirschauer, Kalthoff, and Nieswand (2013) recall. Hence, this method is particularly important in cases where researchers either cannot find a (mental, physical and/or material) way out of the field (see also Blagté, 2014), tend to continually exoticize and/or idealize the field or have difficulties to reaching a point of fieldwork saturation. In most of these cases, fieldworkers develop an affective relation with their field and its actors, and methods for coping with emotions through individual and shared reflexivity are much-needed – even in connection with a field one does not like ( Avanza, 2008).

The most striking effects of field-crossing occur when the field-crosser has little knowledge of her colleague’s field, as I wrote in a book I co-authored with my Japanese colleague ( Salzbrunn & Sekine, 2011):

From our first to last meetings in Paris, I took Prof. Sekine to the empirical field in which I have been doing fieldwork for 12 years, the Parisian district of Sainte Marthe. These common trips were a deep source of mutual discoveries, an opportunity to turn a familiar, common field into a gift box full of the unexpected: thanks to Prof. Sekine’s viewpoint on the houses, people dwelling in the streets, and to the contacts he made on the street because of his perceived otherness, I rediscovered the field in which I had shifted my position from being a newcomer and a stranger to being a part of the neighbourhood, a friend of some people, and an active citizen, conducting participant observation on local political struggles and festive events within that space, finally transforming this formerly unknown place into part of my lifeworld (in the sense of A. Schütz). Thanks to my new alienation in the company of a newcomer, a visitor, I could practice once again the “Befremdung der eigenen Kultur”/alienation of one’s own culture ( Hirschauer/Amann, 1997), an epistemological approach that enables researchers – especially specialists initially trained for researching “exotic” societies -- to pursue anthropology at home. ( Salzbrunn, 2011: 81-82).

In ‘Anthropology as Cultural Critique’, George E. Marcus’ and Michael J. Fischer’s (1986: 137) concept of “defamiliarization” also aimed at disrupting “the common sense, doing the unexpected, placing familiar subjects in unfamiliar, or even shocking contexts”. During field-crossing, cultural elements familiar to the main researcher might be unfamiliar, shocking or disturbing for the field-crosser, who thus helps defamiliarize the main researcher with her field. It is also possible to enter the field as a field-crosser, without being introduced, and to later elaborate on different perceptions and questions during common stays with the main researcher within the field. Another option is to send a field-crosser who grew up in the cultural environment of the main researcher’s field but who specialized in different empirical fields and/or subjects of research to join her/him. The field-crosser might then (re)discover a seemingly familiar country or milieu from an anthropological point of view through the analysis of the main researcher. The latter might be enriched by the implicit understanding of cultural codes possessed by the field-crosser.

During my two stays as an invited research professor at Japan Women’s University Tokyo and at Kwansei Gakuin University in Japan, I took on the Benjaminian role of a performative flaneur ( Köpping, 2005), sometimes alone and sometimes accompanied. I was taken by my colleagues Yasumasa Sekine ^1^, Tom Gill, and Masako Kudo and their students to their respective fields, reacting to the spatial exclusion of the homeless, Korean immigrants’ working conditions, hidden gambling, and prostitution areas, “with my naïve questions, observations and surprises” ( Salzbrunn, 2011:82). Even the use, occupation, and appropriation of seemingly banal places like playgrounds in a context of neo-liberal urban transformation turned out to be a mirror of glocalised political and economic power relations. I visited the latter without my colleagues, but shared my impression in their research seminar, providing new insights about the neoliberal transformation of cities and the appropriation of public space through the observation of these places through an unfamiliar perspective ^2^.

## Methods

Developing my ideas, first published in Salzbrunn, 2011 (pp. 81–84) and tested in teaching as well as in various research projects, I define the principal benefits of field-crossing as the following:

1. Access a new, unfamiliar field thanks to an introduction from a colleague.
2. Accompany your colleagues into their fields so they may benefit from your surprises, remarks, emotions, impressions.
3. Walk through the entire space covered by the empirical field in question in order to get acquainted with the context and speak about your observations and perceptions with your colleague.
4. Revisit your own field through the lenses of your colleagues, unfamiliar with the field and the environment.
5. Develop new, unexpected scales and levels of comparison which enable you to conceptualize common points and differences.
6. Shed light on a common research interest or subject from different angles and perspectives.
7. Reflect on your position within the field.
8. As a field-crosser, help your colleagues reflect on their positionality within the field.
9. As a main researcher, step emotionally and physically out of the field after a long period of immersion and/or apprenticeship.
10. Allow your colleagues to exit the field by helping them to return to a reflexive perspective.
11. Put into question the hegemony of analytical categories by benefiting from your colleagues’ reactions.
12. Contribute to the decolonization of researchers’ prefigurative mental and intellectual settings.
13. Gain coherence in a collective research project by having experienced your colleagues’ field and the way the research topic unfolds in each case.

The recommendation to walk through the field in order to get acquainted with the whole environment and context recalls Roland Girtlers advice to explore the field like a “Wanderforscher” ( Girtler, 2004: 51–54). It is particularly interesting to cross the field by foot during the exploratory phase of a research project:

During the first explorative phase, the following aspects become important:

1. Being aware of emotions, whether uncomfortable or not.
2. Noticing everything which surprises, disturbs, worries, or scandalizes. There is no second chance for a first impression, so intensive writing during this initial phase is paramount.
3. Repeating the exercise from the beginning after a field break and analytic sessions. Noticing everything which surprises, disturbs, worries, or scandalizes. Comparing these impressions with the first notes taken during the exercise.
4. Becoming a stroller, a flaneur. What are the thresholds ( Benjamin, 1982: 618)? Why can these thresholds look like obstacles? How best to overcome them?

Field-crossing could ideally be implemented during or after the explorative phase and the following intensive fieldwork phase. However, these recommendations can be followed in a flexible manner. In some situations, it can be helpful to start fieldwork in a team of two or three researchers, and to carry out a debriefing about different perspectives, viewpoints, perceptions, feelings, information gathered, etc. in the field. At any point, it can be helpful to ask the field-crosser for help in specific challenging situations or in understanding difficult or ambiguous relations within the field. During field-crossing with a colleague, the following questions help to structure the experience:

Leading questions for carrying out field-crossing with a colleague:

1. Where do you want to take your colleague?
2. Why do you want to take them there?
3. What do they notice?
4. How do they feel in these places/during these events?
5. How does their presence change the dynamics in your field?
6. Which of their reactions surprises you?
7. What do you perceive differently?
8. What do you understand differently, according to your perceptions but also according to language abilities?
9. Have you found different expressions of the subject you are studying (in our case art and activism) in the field, and how do they differ?

Drawing on Erickson & Stull’s (1998: 12) assertion that, “Positive personal and professional relations are essential to successful long-term projects”, I also consider these factors important in field-crossing. This includes the capacity to accept different perceptions and views of the same field. During and after field-crossing experiences, it is important to organize feedback sessions on the differences and points of convergence that stand out in field journals and observation protocols. Field-crossing also helps researchers better cope with physically or psychologically difficult situations and to widen their horizons. Referring to contradictory perspectives, the authors propose (1989:4/9):

If several people examine a similar area, the differences in their biases will generate contradictions in their reports. Contradictions, rather than being viewed as threatening, should be seen as the beginning of a better question, a signpost pointing to a more sensitive understanding. Too many potentially rich contradictions get lost in the politeness of academic rhetoric ( Agar, 1996: 99)

It is crucial to remember that the relational nature of ethnography and the impact of personal relations is necessarily implicated in the willingness of people to trust researchers and share their lifeworlds with them. Because the positioning and perception of each researcher impacts relations within the field, and objectivity is impossible to attain in this context, each team member’s subjectivity should be assumed and constantly reflected upon. Reflexivity concerning the researcher’s positionality, honest exchanges on perceived differences with field-crosser’s results, as well as regular feedback sessions with the artivists regarding the research outputs are all the ingredients of solid social anthropological work.

Feedback sessions are particularly important when it comes to the use of images produced by researchers. During field-crossing, each researcher relates differently to the field and its actors. In the ARTIVISM project, the main researcher became part of the field through apprenticeship and multi-sensory ethnography, the field-crossing approach and/or through joining the field occasionally. All these avenues entailed different ways of seeing, hearing, feeling, touching, smelling the field. When each researcher produced images for a collaborative film, she added “layers of reflexivity, as a community gets to see and hear themselves as others have come to see and hear them” (Feld debating with Panopoulos in id. *et al.* 2020: 434). Images taken by researchers have an impact on the artivist’s self-perception. Artivists also might want to use these images to promote their work and/or claims. Furthermore, these images can be used in video-elicitation work, since the debates that unfold during feedback reveal a lot about the ways the artivists perceive themselves (perhaps in contrast to the ways the main researcher and field-crosser(s) represent them). Even though the “co-construction of images” ( Salzbrunn, 2020) is part of our research philosophy, there can obviously be a difference between constructing images of oneself, the other, alone, and together.

The recommendations mentioned above are also valuable for digital team ethnography, as developed by Beneito-Montagut, Begueria, and Cassián (2017): Senior and junior researchers can adapt different roles within a clearly defined hierarchical division of labour, discussing “different interpretations and perspectives in the observation of the same situation” (2017:667). The complexity of meaning can better unfold through a collective process and common discussions.

### Field-crossing in the ERC ARTIVISM project

In the following section, I provide an example for field-crossing applied during the conduct of my ERC ARTIVISM project. As mentioned above, the project focuses on “Art and activism. Creativity and Performance as Subversive Forms of Political Expression in Super-Diverse Cities” and seeks to “explore new artistic forms of political expression under difficult, precarious and/or oppressive conditions” ( Salzbrunn, 2015:1). We have been doing fieldwork on three continents, with one researcher investigating a main field and the colleagues joining her as field-crossers. The fields include: comic art in Cameroon and Europe, official and independent carnivals and carnivalesque political performances in (Mediterranean) Europe, and (mostly Chicana) mural art in California. The senior researcher, Raphaela von Weichs, had taken the principal investigator (PI) Monika Salzbrunn and one of the PhD students, Federica Moretti, to the MBOA BD (comic) Festival in Douala and Yaoundé. The PI was joined in her Italian fields (one year in Genova, but also shorter stays in Viareggio and Florence) by the team members in various constellations: PI and senior, PI and PhD student Sara Wiederkehr, PI and technician for filming, PI and/or technician, and PhD student and/or senior. The technician, who had his own personal network of friends engaged in artivist circles and actions, also took images alone in Genova and Marseille. In the latter field, he was joined by the PhD student and/or the PI. The whole team also did field-crossing in Nice where Federica Moretti was based for one year.

Each team member had a different knowledge basis in each context with regards to political situations, spoken languages, and personal connections. Her previous knowledge influenced the way she approached the field, perceived social dynamics, and understood artivist practices. The PI sometimes had easier access to institutions due to her authority, but her presence could also have an impact on certain people sensitive to hierarchy. Nevertheless, a PI might be more familiar with certain political milieus and therefore more at ease than a PhD student who does not feel comfortable with certain actors or situations. A team member who is not fluent in the language of the fieldsite can take advantage of their multiple senses and perceive multi-sensory aspects of the field and its social relations through smelling, hearing, touching, viewing, tasting, walking, etc. They can also communicate differently and might find interlocutors in the field who are multi-lingual and open other, unexpected doors.

In summary, field-crossing as a collaborative method contributes immensely to:

1.   Create spaces of exchange between interdisciplinary researchers who are participating in the project

2.   Create spaces of exchange between the ethnographic fields studied in the project

3.   Create spaces of exchange between the analytical core-concepts of the project as they are applied to each field ( Salzbrunn, 2015:8–9).

Each researcher has different knowledge regarding the various art forms we have been researching, and each individual reacts differently to public events like the MBOA BD Festival in Doula and Yaoundé, the official carnivals in Nice and Viareggio, and the independent, anarchic carnivalesque events in Genoa, Florence, Marseille, and Nice. The latter are particularly interesting and challenging: a large crowd behaves in a relatively free way across a wide space, since these events are enacted as a deliberate counterpart to the extremely strict and controlled top-down official events. Each researcher’s perceptions and interpretations during these anarchic, disruptive and/or risky events within the event (e.g., a big fire led at night as a closing ritual) is a particularly rich source of controversial debate and analysis.

Besides the regularly planned field-trips, additional, spontaneous field-trips are organized during the whole project, according to specific events occurring in each field.

During the (ongoing) last phase, follow-up fieldwork has been conducted by the team, each researcher alone and several sub-teams together, according to the opportunities, availabilities and access to the events. In each field, the main researcher and field-crossers have provided images, montages, and work-in-progress films in order to gain feedback and to modify the editing according to the people depicted and the extent to which they agreed to the use of their images.

### Challenges and problems

Fieldwork is not only the most exciting part of social science research, but also, as Girtler ( Girtler, 2001: 85) underlines, physically and psychologically demanding. This can particularly be the case in a field a researcher does not feel familiar or at ease with. Misunderstandings can also occur in highly changing and dynamic groups, when the roles of field-crossers are not clear or comprehensible to each person in the field, and when the field-crosser needs to explain her presence several times while talking to different interlocutors. Such situations afford adaptations and strategies on the part of the field-crosser in order to become embedded in the field (e.g., through the experience of a short-term apprenticeship). Here, the possibility to reflect together on the reasons for rejection or uncomfortable situations helps researchers cope with the situation in situ, and to benefit from it during further analysis.

The opposite situation can also occur: What happens if someone gets so deeply immersed in the field that they ‘go native’ and lose their distance to the research subject, whether mentally, intellectually, or physically? This problem has been much debated in classical anthropology, with the concept of reflexivity coming fore as a remedy. However, self-reflection has its limits. After one year as an apprentice, strong ties can lead to conflicts of interest surrounding findings, their interpretation, and the question of what content should or should not be published. Restrictions in publications are also related to ethical issues, so that a balance must be found between respect for the artivists’ points of view and interests on the one hand, and the researcher’s perspective on the other, which should go far beyond simply reiterating the artivists’ declarations. During this phase, field-crossing is particularly helpful since the field-crosser might notice these conflicts of interest, contradictions, individual, mutual, and collective pressure and risks for each party. Conflicts and other difficult and painful situations are to be taken seriously and need to be individually and collectively analyzed, as they reflect the complexity of what is/was going on in the field, and because this debate helps to regain reflexivity. If the artivists finally disagree or reject certain interpretations provided in publication drafts or draft montages of films, this also poses a challenge but is often particularly revealing to reflect on as well.

What happens if a team member of the research project completely shifts roles, from technician to activist? As fieldwork always goes along with personal, emotional, and physical implications, – especially during an apprenticeship – this experience has an impact on each person who is part of it, whatever role they have taken on. It can happen, for example, that a video technician, hired for filming a specific field site, identifies more and more with the place and creates strong links with the research subjects to the degree that they become an activist and/or gatekeeper in turn. In that case, the reflection on this shift is part of the research process and an integral part of the findings to be analyzed. Field-crossers who might be less implicated can also help to negotiate and solve the conflict of interest that arises from this shift of roles.

## Conclusion

This article shows how field-crossing helps researchers reflect upon their positioning in a field, open new perspectives, integrate surprises (serendipity), disruptive and unexpected turns of events, and cope with inner and collective conflicts. A general condition for field-crossing is an openness to surprise, irritation, potential discomfort, and excitement elicited by ethnographic findings, ideally based on apprenticeship and a multi-sensory approach. The latter requires a commitment to diving physically and mentally into a field (for one year if logistically and personally possible), and an openness towards processing the impact of this field-experience on oneself and one’s personal relations. Field-crossing helps researchers leave the field mentally and physically, to regain reflexivity and the necessary distance in order to analyze the findings and social dynamics occurring within the field, and to solve potential conflicts of interest between the main researcher, the team members, and the artivists. The perceptions of the field-crossers broaden the main researcher’s horizon and increase the field-crosser’s understanding of their colleagues’ fields and highlight aspects which have become so familiar that they may be overlooked. Taking a fresher perspective, the field-crosser might have a better view of interpersonal dynamics and conflicts occurring in the main researchers’ field, and can contribute to solving them, or at least gaining distance from them. Social roles, age, gender, origins, ways of living, family relations etc. all have an impact on the relations one can create within a field. The integration of different field-crossers adds variation in terms of field access and relations, which in turn enriches access to different aspects of the field.

Finally, feedback sessions with the artivists about text and image publications (e.g., comics, films) can also serve as elicitation sessions which reflect the complexity of field relations and individual and collective perspectives. Ethnographers must go beyond a simple restitution of viewpoints and analyze complex political issues. But as the latter potentially implies conflicts of interest, regular feedback sessions within the team and contrasting the perspectives of the main researcher and the field-crossers helps gain the necessary distance to overcome these challenges constructively.

## Data availability

No data are associated with this article.

## Footnotes

^1^ Comparison, of course, has always guided Prof. Yasumasa Sekine’s and my work (see Sekine, 2006; Salzbrunn, 2008). But here, we managed to go one step further by sharing our observations, finding common points and differences, observing a common field with new lenses.

^2^ Salzbrunn, Monika. “The street as a mirror of society. Field impressions from Tokyo and Kyoto.” Graduate seminar of Prof. Yasumasa Sekine at Kwansei Gakuin University, Japan, May 22nd 2014. A comparative view on thresholds and urban gardening was published in Japanese: Salzbrunn Monika, 2018: パリと東京の都市の路上で共通。芸術、音楽、都市ガーデニングを通じて通りを充当 (“Commonality on the urban street in Paris and Tokyo. Appropriating the street through arts, music and urban gardening”), in: Yasumasa Sekine (Ed.), ストリート人類学 (Street Anthropology), p. 158–178, Tokyo.
