# Supplementary material for: Researching and practicing ARTIVISM through field-crossing: an innovative method for collaborative research
Source: Open Res Eur. 2021 Sep 13;1:106. [Version 1] doi: 10.12688/openreseurope.13634.1 (PMC10445878; doi:10.12688/openreseurope.13634.1)
Supplement: Peer review history

# Peer review: Reviewer response for version 1

**DOI:** 10.21956/openreseurope.14705.r28441

**Published:** 2022-02-23

**Authors:** Paula Serafini

**Affiliations:** 1 Queen Mary University of London, London, UK

While it is widely acknowledged that researchers must engage in reflexivity during fieldwork, often the matter of how this is done (and how this is done well) is taken for granted. This paper addresses this issue by offering a method for embedding and sustaining reflexivity in collaborative research.

 To begin with, the paper discusses some of the main issues related to reflexivity. These include the opportunities and challenges associated with being an insider vs an outsider researcher, the impact of positive (or negative) emotions towards the field of research, and strategies for defamiliarizing oneself with the field, all issues that have had a central role in fields like anthropology and feminist geography for a while now (e.g. Nagar & Geiger 2007 ^1^, Sultana 2007 ^2^).

 The original contribution of the paper is the method proposed, which the author names ‘field-crossing’, and which consists of inviting other researchers to join the lead researcher in the field to aid the process of reflexivity. The author describes different possibilities for field-crossing between researchers with varying degrees and forms of knowledge of a field, which include experts, researchers with no background knowledge, and those who might have a background familiarity with a context (e.g. due to having grown up in the place in question), but whose academic work is on a different area. When a researcher with a different degree and/or form of familiarity joins another researcher that is currently in the field, the incoming researcher can provide their first impressions and insights. This is valuable knowledge that allows the lead researcher to gain a refreshed perspective on their field, activating in this way a process of reflexivity. At the heart of this method is a call to embrace the subjectivity of the researcher’s perspective as a way to help them contextualise their findings. This contextualisation, a sort of ‘locating’ of the researcher’s perspective on a given matter, then allows the researcher to better understand the meaning of their contribution as part of a wider whole: in other words, the limitations of their contribution, but at the same time, the specificity and originality.

 In just a few pages the author manages to clearly present an innovative method that makes an important contribution to the practice of reflexivity. It would have been good, however, to see some more concrete examples of what field-crossing can achieve. Something else to consider is that the paper argues that field-crossing contributes to “the decolonization of researchers’ prefigurative mental and intellectual settings.” However, there is no explanation of how this is achieved.

 The author acknowledges questions of authority and power and how these affect the experience of the field for different researchers in a team. Towards the end of the article, she also states that “Social roles, age, gender, origins, ways of living, family relations, etc. all have an impact on the relations one can create within a field. The integration of different field-crossers adds variation in terms of field access and relations, which in turn enriches access to different aspects of the field.” (p.9) What would have been good to also see is some discussion of the potential barriers and challenges faced by researchers with different gender, sexual, racial, and ethnic identities and dis/ability when crossing fields. The ability to approach the field as a flâneur, for instance, can vary greatly for different people, as pointed out by  Gibbons (2015) ^3^ and Sharanya (2016) ^4^ (I also discuss this matter in relation to psychogeography in the UK, see Serafini 2018 ^5^).

 Overall, this paper successfully presents an innovative and exciting method that responds to an important practical and ethical question, and it also opens up many avenues for further enquiry. I look forward to reading more about field-crossing and about this project’s findings.

Are sufficient details provided to allow replication of the method development and its use by others?

Yes

Is the description of the method technically sound?

Yes

Are the conclusions about the method and its performance adequately supported by the findings presented in the article?

Partly

Is the rationale for developing the new method (or application) clearly explained

Yes

If any results are presented, are all the source data underlying the results available to ensure full reproducibility?

Yes

Reviewer Expertise:

Art activism, cultural politics, extractivism, environment, performance, social movements, creative industries, cultural policy, ethics of care

I confirm that I have read this submission and believe that I have an appropriate level of expertise to confirm that it is of an acceptable scientific standard.

## References

[1] : Reflexivity and positionality in feminist fieldwork revisited. *Politics and Practice in Economic Geography. Sage Publications Inc* .2007; 10.4135/9781446212240.n22 267-278 10.4135/9781446212240.n22

[2] : Reflexivity, positionality and participatory ethics: Negotiating fieldwork dilemmas in international research. *ACME: An International Journal for Critical Geographies* .2007;6(3) :374-385 Reference source

[3] : Salvaging Situationism: Race and Space. *Salvage* .2015; Reference source

[4] Walking the Walled City. Gender and the Dérive as Urban Ethnography. authors. *Etnoloska Tribina; Zagreb* .2016;46(39) : 10.15378/1848-9540.2016.39.09 189-212 10.15378/1848-9540.2016.39.09

[5] : Performance Action. The Politics of Art Activism. *London: Routledge* .2018;

# Peer review: Reviewer response for version 1

**DOI:** 10.21956/openreseurope.14705.r28295

**Published:** 2022-02-10

**Authors:** Carolyne Ali-Khan

**Affiliations:** 1 University of North Florida, Jacksonville, USA

I love this paper. It is conceptually sound, methodologically innovative, fresh in its approach, well theorized, and interesting. It sheds new light on some fascinating research techniques. It is clearly delineated to help replicability. I look forward to seeing it in pass peer review. I have only a few small suggestions.

 There are some places where some of the ideas in the text would be more clear to readers if they were spelled out a little bit. Particularly in the introduction. For example within a few sentences we learn, “The event approach ( Salzbrunn, 2021), applied to the research of artivism, is a constructive answer to groupism ( Brubaker, 2004).” Although some readers may be familiar with the event approach and constructive answers to groupism others like myself will be somewhat perplexed by this. I assumed that it would be spelled out later but to do so in the introduction would be helpful to comprehension, and keeping readers reading.

 Similarly later in the article the authors state “Field-crossing helped maintain a balance between the long-debated risks of “nostrification” (following a Malinowski-style immersion) and “othering” ( Said, 1978)” I am (and I suspect most readers of this journal are familiar with “othering” however nostrification was new to me. I think the authors might be helped by a dose of their own technique, a non-Ceasar read with subsequent clarifications added might help the strength of the text.

 Sometimes the language is a bit obtuse but in other places the wisdom and clarity of the researchers really shines. This is particularly true in the Caesar/non-Caesars discussion. And as the paper progresses it becomes more fluid and easier to read. The “how and why to” bullets are really helpful. Overall, the article makes a strong and important argument for a new methodological collaborative approach, one that has the potential to really bring in the strengths of collaboration in powerful ways. This article also contributes to pushback against a body of literature that is often reductionist in that it is non-sensory. Reading this made me even more aware of the shortcomings of paradigms that reduce collaboration to pure instrumentalism when it can be so much more. Kudos to the authors. This offers an important contribution to the literature.

Are sufficient details provided to allow replication of the method development and its use by others?

Yes

Is the description of the method technically sound?

Yes

Are the conclusions about the method and its performance adequately supported by the findings presented in the article?

Yes

Is the rationale for developing the new method (or application) clearly explained

Yes

If any results are presented, are all the source data underlying the results available to ensure full reproducibility?

Yes

Reviewer Expertise:

Sociocultural contexts in education, qualitative research, arts based pedagogies, critical pedagogy.

I confirm that I have read this submission and believe that I have an appropriate level of expertise to confirm that it is of an acceptable scientific standard.

# Peer review: Reviewer response for version 1

**DOI:** 10.21956/openreseurope.14705.r28005

**Published:** 2022-01-20

**Authors:** Daniel Bertaux

**Affiliations:** 1 Laboratoire Dynamique Européennes, Université de Strasbourg, Strasbourg, France

This is an article presenting a new *method* for fieldwork research, a method which the author calls "field-crossing". Simply said, it consists in inviting an anthropologist from another culture to join when and where you are doing fieldwork; Monika Salzbrunn did that with Professor Sekine, a Japanese anthropologist (he went with her to some of the places she had done fieldwork, and she went with him in some of his own ones in Japanese cities). The general idea is that this colleague, being a total outsider, will observe and interact with a completely fresh eye, thus helping you to partly compensate your own loss of freshness as you become more and more familiar with a particular field.

 This idea should be explored thoroughly, says the Abstract: lived experiences of doing fieldwork together need to be analysed jointly in "collective feedback sessions (…) serving as elicitation sessions which reflect the complexity of field relations"; adding that some of the main actors from the field itself should participate in such sessions.

 In such circumstances, complexity necessarily moves to some higher order. To the author however, this is not a drawback but a positive characteristic. Articles on methods and "methodology" may often lean on the boring side; but surely not this one! It pulls strings of different colours together - the object/subjects of the research (here artivists), the researchers' affects, their changing perceptions as fieldwork moves forward, interactions, risks, reflexivity… - in a kind of dynamic kaleidoscope that resolutely stands in frontal opposition to principles that previous generations of researchers had taken for granted: don't be subjective, don't go native, try to forget your values and control your affects while doing fieldwork and analysing your data; and don’t give up an inch on the monopoly of social sciences to scientific truth.

 In the history of French sociology such principles were remarkably knit together and strongly expressed in Bourdieu, Passeron, and Chamboredon's *The Craft of Sociology* (1968 for the first, French edition). This book shaped the minds of young French sociologists for several decades. One of its sentences summarizes the (scientist) faith and spirit of its authors as they were writing: "C'est peut-être la malédiction des sciences de l'homme que d'avoir affaire à *un objet qui parle*" (The curse of human sciences is perhaps to have to deal with *an object that talks; *p.64, italics in original). While some features of the epistemological orientation proposed by Monika Salzbrunn seem to run in exactly the opposite direction, her "field-crossing" new method aims ultimately at the very same goal: to move knowledge forward. But she herself makes the point that while the goal is the same, the approach she proposes is entirely new : "Taking into consideration how fieldwork affects each individual via field-crossing is part of the affective turn in social sciences (after a long period influenced by positivism and scientism) and contributes to the further development of multi-sensory anthropology."

 If she had given concrete examples of how field-crossing has led to new fieldwork discoveries, it would have helped readers to better understand her sophisticated arguments (which however are backed by a robust list of references). Perhaps, as one of the four co-founders of Migreval - the collaborative French-German research project she mentions at the beginning of her paper - I can offer one. Migreval research project is mostly built on the collection of life stories of non-EU "irregular" (i.e. undocumented) migrants having arrived and settled in either Strasbourg or Frankfurt-am-Main at some point during the last thirty years. While interviewees are at first left totally free to develop their own narratives, interviewers keep in mind that as researchers they are primarily interested in reconstructing with as much precision as possible the first days, weeks and nths in the host country (here, respectively, France or Germany) on the assumption that what happened to any newcomer during these first moments - interactions, new encounters, information gathered, events… - proved very important in shaping his/her subsequent life course, as well as those of family members eventually accompanying him/her. Also, interviewers remain aware that Migreval is ultimately about providing evaluations of (national, regional, and local i.e. city-level) *policies* towards migrants: not top-down evaluations based on social statistics, but bottom-up evaluations based on multiple "individual" (or rather, family) case histories.

 One of the unexpected discoveries of the Migreval research project - which is still going on - has been precisely the importance, for undocumented migrants, of the *very first encounters* they had with members of the host society. Most of such encounters happened by chance; they could as well have never happened. Their consequences, in the long run, proved however considerable.

 When they finally arrived in Strasbourg or Frankfurt from Syria, Iraq, Afghanistan, or Sudan, most undocumented migrants had no place to stay. They did not speak the host society's language and had no idea whatsoever of the range of possible initiatives they could take (with the help, for instance, of some local voluntary association) to avoid being deported. Of course, they were desperate to find some fellow countryman with whom they could communicate and who hopefully could help them meeting their most urgent needs, as well as those of their children and other family members accompanying them. But to begin with, where to find them in the new urban environment they had just landed in? In most European cities, non-EU migrants live and work in some peripheric area, whereas newcomers usually arrive by train in the central, "downtown" one. Thus, newcomers who do not speak the host society's language and who are afraid of being arrested by the police may remain in semi-hiding for days and weeks. Some others however having exchanged a smile with a passer-by, may start a 'conversation' using their hands and face, thus starting a long process towards integration.

 Indeed, city dwellers in Frankfurt or Strasbourg are rather well-disposed towards migrants, much more anyway than their respective governments: after a first eye-contact and exchange of smiles, it often happens - as life stories stored in the Migreval database show - that the city dweller will offer some help; the more so if the adult migrant has a child or baby with her/him. He or she might even be given temporary shelter in some home; or at the very least the phone number of some voluntary association known for helping undocumented migrants.

 Monika Salzbrunn righty insists on the importance of taking into account the overall *context* if one wants to fully understand what is happening at a much smaller scale within it. This is a fully justified general principle that may be further specified: while "the context" of a given phenomenon under study is always very wide and differentiated, what is *relevant* for the researcher are only those of its numerous elements that do interact with that phenomenon.

 Here is another example, also taken from the Migreval research project. It has been initiated by four sociologists, two German ones (Ursula Apitzsch and Lena Inowlocki) and two French ones (Catherine Delcroix and Daniel Bertaux). The first three researchers have been studying international migrations for decades, unlike myself who is therefore sort of cast in the role of "field-crosser". Among the numerous phenomena that we were getting at through collecting life stories and studying policies towards migrants, there was this pattern: while State authorities (governments) multiplied statements against illegal migrations, about border closure and the necessity to "preserve national identity", lower-level authorities and especially city-level ones seemed much more open to welcoming illegal migrants and helping them settle in "their" city. This pattern could be observed in various places, and even in configurations where a liberal government appeared in practice less hostile to illegal migrants' settlement than local, city-level authorities (mayors) elected by conservative or even reactionary electoral majorities. Why could it be so?

 Given that I was not a specialist of immigration issues, I looked at actual practices. For undocumented migrants, whether they come from Russia, Ukraine, Turkey, or the Middle East, who succeed in entering the Central European "Schengen area", Strasbourg - where we live - is the door to France, and a likely choice to settle. An interesting point is that Strasbourg's successive mayors have been keen to mention that their city is one the founders and leaders of the European network of Welcoming Cities ^^[1]^^. Why is that so that, *independently of their political orientation, *they seem so keen to help migrants to settle in the city they govern?

 When reaching a European city, undocumented migrants have initially no choice but to sleep in its parks, living in its streets and (most of the time) spending the day begging near the central square. Most passers-by do not know anything about why these people, who don’t speak French, got here. But since they seem to be homeless, it means the mayor has not been able to take good care of them. Passers-by will take it as shaming their city; some might even make a scandal about it.

 And it is precisely to avoid this kind of politically damaging development that mayors - whatever their political orientation, whether they like it or not: this is the point - will take the necessary steps to find temporary housing for undocumented migrants. They will even try providing them jobs to keep them from begging in 'their' streets ^^[2]^^. 

 As a text presenting a new method of doing fieldwork (a method that author calls "field-crossing"), the text is clear; it lists a number of properties of this new method, so that as a methodology text it is self-sufficient, well-written and deserves to be indexed as such. Therefore: **Approved.**

 However, as it now stands, the text lacks concrete examples of field-crossing. Which is all the more frustrating since the author says she has experimented it for already ten years in a number of fieldworks researches (and this is certainly true). So, why not choose at least one, or two or even three concrete examples of the added value of field-crossing? Examples of (creative and useful) original obsevations, new intuitions, new hypotheses, new ideas which were expressed by a newcomer colleague  (e g a Japanese social anthroplogist)  invited by the author to come and visit the place where she was doing fieldwork. Or alternatively, original observations, intuitions, new hypotheses, new ideas that came to the mind of the author herself when visiting for the first time the "field" of some colleague.

 This is what I think of her text. Without examples it is a good, well-written and creative paper which brings to light a new method. I have no reservations neither on its form nor on its contents. As it stands, the text (which obviously is the result of considerable work, including the work of finding its definitive written form) deserves already the status of Approved as a methodology text.

 It remains, however, for lack of concrete examples, somehow on the abstract side. With a few well-chosen examples added, this same text would communicate much better the substance and usefulness of the new method. The Author's argument would become (much) more convincing - which, at the end of the day, is exactly what she wants.

 [1] "In 2015, Strasbourg launched a European Network of Solidarity Cities with its partner cities of Catania and Rovereo, with the aim of developing initiatives to ensure that refugees can be welcomed and integrated in a dignified manner." https://www.coe.int/en/web/interculturalcities/-/un-reseau-europeen-de-villes-solidaires

 [2] Even more surprising, Iraqi Christian families in great danger of extermination by Daesh found refuge, through a priest, in some Alsatian villages where they were warmly welcome. See D. Bertaux and S.-A. Bevilacqua, "Asylum Seekers's Reception in Western European Villages: Spatial Proximity as a Key to Integration", submitted to *European Societies.*

Are sufficient details provided to allow replication of the method development and its use by others?

Yes

Is the description of the method technically sound?

Yes

Are the conclusions about the method and its performance adequately supported by the findings presented in the article?

Yes

Is the rationale for developing the new method (or application) clearly explained

Yes

If any results are presented, are all the source data underlying the results available to ensure full reproducibility?

Yes

Reviewer Expertise:

Sociology

I confirm that I have read this submission and believe that I have an appropriate level of expertise to confirm that it is of an acceptable scientific standard.
